# The ubiquity of selective attention in the processing of feedback during category learning

**DOI:** 10.1371/journal.pone.0259517

**Published:** 2021-12-16

**Authors:** Katerina Dolguikh, Tyrus Tracey, Mark R. Blair

**Affiliations:** 1 Cognitive Science Program, Simon Fraser University, Burnaby, Canada; 2 Department of Psychology, Simon Fraser University, Burnaby, Canada; University of Sydney, AUSTRALIA

## Abstract

Feedback is essential for many kinds of learning, but the cognitive processes involved in learning from feedback are unclear. Models of category learning incorporate selective attention to stimulus features while generating a response, but during the feedback phase of an experiment, it is assumed that participants receive complete information about stimulus features as well as the correct category. The present work looks at eye tracking data from six category learning datasets covering a variety of category complexities and types. We find that selective attention to task-relevant information is pervasive throughout feedback processing, suggesting a role for selective attention in memory encoding of category exemplars. We also find that error trials elicit additional stimulus processing during the feedback phase. Finally, our data reveal that participants increasingly skip the processing of feedback altogether. At the broadest level, these three findings reveal that selective attention is ubiquitous throughout the entire category learning task, functioning to emphasize the importance of certain stimulus features, the helpfulness of extra stimulus encoding during times of uncertainty, and the superfluousness of feedback once one has learned the task. We discuss the implications of our findings for modelling efforts in category learning from the perspective of researchers trying to capture the full dynamic interaction of selective attention and learning, as well as for researchers focused on other issues, such as category representation, whose work only requires simplifications that do a reasonable job of capturing learning.

## Introduction

Research into the cognitive processes that enable a human to classify objects into categories has a long history within psychology (e.g., [1]). Work in this field has produced a variety of computational models that describe how mental representations of various kinds (e.g., prototypes [2–4], stimulus exemplars (e.g., [5, 6]), or stimulus clusters [7]) can be used to predict how someone might classify a particular stimulus. Early models were aimed at capturing asymptotic performance, and thus might be called categorization models. Later models emphasized understanding and predicting the entire learning process. These category learning models use learning algorithms, such as backpropagation, to adjust connections between representations of stimuli or stimulus features and categories [8], and also to weigh (or attend to) various stimulus dimensions [9]. In all these models, knowing the correct category is crucial to the learned association of features to categories, regardless of differences in how categories might be represented. Thus, these models instantiate a simple and uncontroversial idea: feedback is crucial to learning.

Research in category learning tends to use a prototypical experimental procedure. In the response phase, a stimulus is presented for classification into one of several possible categories. The stimulus has features that vary along different dimensions and from which the correct category can be derived. For example, stimuli might be cartoon bugs that could have one of two different types of antennae, body shape, feet, and eyes; category A bugs might tend to have round bodies and long antennae, and category B the opposite. After the participant inspects the various features and responds with a category decision, the feedback phase begins. The correct category is indicated (appearing in one of the corners of the screen, for example) and the stimulus is re-presented. After that, the next trial begins with a new stimulus. An experiment might have a few hundred trials, during which it is hoped that most participants will learn how to classify all the stimuli correctly. It is common for both the response phase and the feedback phase to be self-paced; that is, the participants themselves decide when they are ready to select a category, and for how long to view feedback before moving on the next trial.

It seems clear that specific feedback, in the form of the correct category assignment for that stimulus, enhances our ability to learn. How this works in category learning research is still unknown, and because the primary topic of interest in the field is category representation, there is little research into the mechanics of feedback processing. For the response phase of the experiment, wherein participants are deciding what category to choose for each stimulus, work has been done to understand reaction times [10] and how attention is allocated to stimulus features [11–13]. However, few studies even report analogous feedback phase data, much less taking them as a target of investigation (for an exception, see [14]).

Despite the general lack of attention-based research focused on the feedback phase, there is evidence from several sources to suggest that learning is influenced by the manner in which participants experience feedback, and thus some reason to think that theories of category learning can be improved by being extended to account for feedback processing. Several studies suggest that allowing participants more time between trials improves learning. Bourne and Bunderson manipulated the inter-trial interval (ITI)—the time between the end of one trial and the beginning of the next—and found effects on learning, with the optimal ITI 9 seconds long [15]. In a later experiment, Bourne and colleagues found that maintaining the stimulus onscreen while presenting feedback resulted in better learning than removing the stimulus [16].

More recently, Watson and Blair used eye tracking to investigate participants’ gaze while processing feedback [17]. In two experiments, participants learned complex categories with self-paced feedback. They found that participants spent more of their time looking at the stimulus features than the feedback signal itself. Further, they found that the time spent looking at stimulus features during feedback on incorrect trials was greater for those participants who were able to meet the learning criterion than for those who were not.

In addition to evidence showing that participants’ attention to a re-presented stimulus is important, recent work has shown that feedback processing might be different for different kinds of categories. Worthy and colleagues investigated effects of stimulus offset (when the stimulus disappears) and delayed feedback onset in category learning [18]. They compared a rule-based categorization structure to an information integration one. In rule-based category structures, simple, verbalizable rules (based on some particular feature of the stimulus, such as size) distinguish the categories. In an information-integration structure, there are no such simple rules because the information from multiple dimensions must be integrated together. They found that if the stimulus was not shown during feedback, performance was impacted for information-integration structures, but not for rule-based structures. Experiments from Maddox and colleagues as well as Smith and colleagues found concurring results; information-integration structures are impaired from delayed feedback compared to rule-based structures [19, 20].

The learning implications from the absence of feedback were found to also change based on category structure. By removing the feedback element from a category learning experiment, Ashby and colleagues found that subjects tended to default to unidimensional strategies, regardless of whether they were assigned structures that required processing of two dimensions [21]. These strategies eventually transitioned into a more optimal unidimensional strategy for basic rule-based structures, but were not effective in discriminating between categories in a information-integration structure.

In a study of the neurophysiological basis of the interactions between learning and attention that used a paradigm very similar to category learning, Leong and colleagues found that a model of participant performance was improved by taking into account participants’ processing of feedback [22]. They reported that eye movements during the feedback phase and during the response phase were independently predictive of task performance, and that feedback-related eye movements provided additional useful information over and above the response eye movements. They also found that as learning progressed, the gaze targets between response and feedback converged, which is especially interesting because attention during response and feedback might easily be imagined to be complementary. For example if the participant looked primarily at Features 1 and 2 pre-response, one can image that participants might spend extra time looking at Feature 3 during feedback, either as a reaction to an error or even just to be sure there was not valuable information. Given that some researchers argue that attention is used as a kind of error correction mechanism (e.g., [8]) this is a plausible prediction. The findings of Leong and colleagues thus point to a heightened theoretical importance of eye movements during feedback early in learning (cf. [17]).

The Leong and colleagues paper [22] also highlights the interest in feedback from work outside the field of category learning. While categorization researchers might justifiably be interested in attention during feedback primarily, or even exclusively, because of its eventual influence on categorization decisions, there are researchers for whom the integration of attention and learning is the primary target of investigation. Researchers like Gottlieb, who advocates for an ‘active sensing’ framework, argue that attention and learning should be studied together [23]. To such researchers, and such modelling efforts, a dataset of eye movements during learning is a valuable resource, and the very aim of their explanatory efforts.

Work on a model integrating learning, attention, and gaze in our lab provides an example of a more integrated approach. LAG-1 is a dynamic neural field theory model of three important systems as they apply to category learning: a category learning system, a spatial attention system, and a saccade timing system, each comprising a set of smaller processing units [24]. The model calculates their mutual influence continuously over time. Based on the associations between features and categories—learned through co-activation of features and categories during the feedback phase—information gain is calculated and drives feature priority and guides targeting of saccades to relevant features. It produces a continuous stream of fixations and responses like a human would. Because the model hypothesizes core cognitive processes and implements them continuously in time, the model is not mute on how feedback will be processed, even though it was not originally designed with feedback, or the present results, in mind. The model predicts that fixations will be guided by information gain, and thus increasingly favour relevant features. It also predicts that the time spent on feedback will decrease with learning as the model more rapidly reaches the threshold due to stronger category-feature associations. In addition to making relatively straightforward predictions about feedback, LAG-1 can, because it is a somewhat generic looker/learner, be applied to other visual cognition tasks, such as visual search. The emphasis is not on category learning in particular, but in predicting how learning and attention interact in real time. A dataset for eye-movements during feedback can directly confirm, or not, the predictions of such a model.

Overall, then, evidence advocates for more study of participants’ allocation of attention while they are given feedback. As most category learning studies do not report data regarding the feedback phase, there is a lack of basic data describing how participants process feedback when learning categories of various types, data which may be of interest to category learning researchers and will be of clear interest for researchers studying the interaction between attention and learning. In the present work, we analyze the feedback phase of six prior category learning studies from our lab. These studies were all originally conducted to answer research questions unrelated to feedback but, because we recorded participants’ gaze data throughout the feedback phase of each trial, they can help us understand feedback processing in more detail while also allowing us to test the generality of prior findings.

Our analysis of the data answers seven questions. First, how long do people spend processing feedback, and how does it change with learning? Second, what do people look at during the feedback phase? Previous research suggests that people spend more time investigating stimulus features than the correct category label [17]; does this generalize to other types of categories? How is attention distributed to stimulus features that are relevant for categorization compared to those that are irrelevant? Third, are there regularities in the order that features are fixated? Fourth, are response phase and feedback phase attentional allocation similar or different, and how do they change with learning? Leong and colleagues found that attention during the response and feedback phases converged [22]—does this replicate and generalize to different kinds of categories? Fifth, do the characteristics of individual gaze fixations change between response and feedback? There is research to suggest that fixation durations decrease slightly with learning [25], and other research to suggest this occurs particularly for incorrect trials [26]. The LAG-1 model generally predicts falling fixation durations because learning will facilitate the rapid reaching of the threshold that triggers a new saccade. While there may be an increased chance that participants will skip feedback, according to LAG-1, there should be no reason for fixations from the response phase to be longer than fixations from the feedback phase. The answer to this question has theoretical relevance for this model, at least. Sixth, is feedback processing different for error trials and correct trials? Seventh, do participants ever ignore feedback entirely? The answers to these questions, across a variety of category types, will provide a good overview of how participants process feedback that will be of use for extending computational theories of attention and learning generally and of category learning; These data will also be useful for researchers constructing experiments using the category learning paradigm, and, more broadly, for researchers interested in feedback and learning from fields such as memory or education.

## Methods

In this paper we investigate important regularities of feedback processing. To do this, we look at the feedback phases of prior category learning experiments from our lab. Where we are using a dataset with results previously published in a journal article, we say so, enabling the interested reader to fit together the two phases (response phase and feedback phase) of the experiments. None of the feedback processing data presented here have been published previously. Because we are in part interested in how the regularities of feedback processing change with different categories and experimental procedures, we group not by experiment, but by measure, showing all the datasets together for comparison. The following is an overview of the datasets used in our analyses. For all datasets, the research was approved by the Simon Fraser University Research Ethics Board. Informed, written consent was obtained for all participants.

### Datasets

#### Dataset 1: Relevance differences across categories, 4 categories (RelDif-4cat)

Dataset 1 was originally from a category learning experiment that investigated the relationship between working memory span and the allocation of attention in category learning. Participants in this experiment were taught categories that used the category-specific feature relevance category structure first used by Blair and colleagues [11]. Some of the data from the category response portion of this experiment were previously reported by McColeman and colleagues as Dataset 8 [25], but the data reported here regarding the processing of feedback have not been previously published. Overall, this category structure was difficult for participants to learn, and most participants displayed a pattern of attention that was different for different categories.

*Participants*. Participants were 220 undergraduates at Simon Fraser University recruited from introductory psychology classes. All had normal or corrected-to-normal vision. Of these, 16 were excluded from analysis for random responding. This left 204 participants included in analysis.

**Fig 1:**
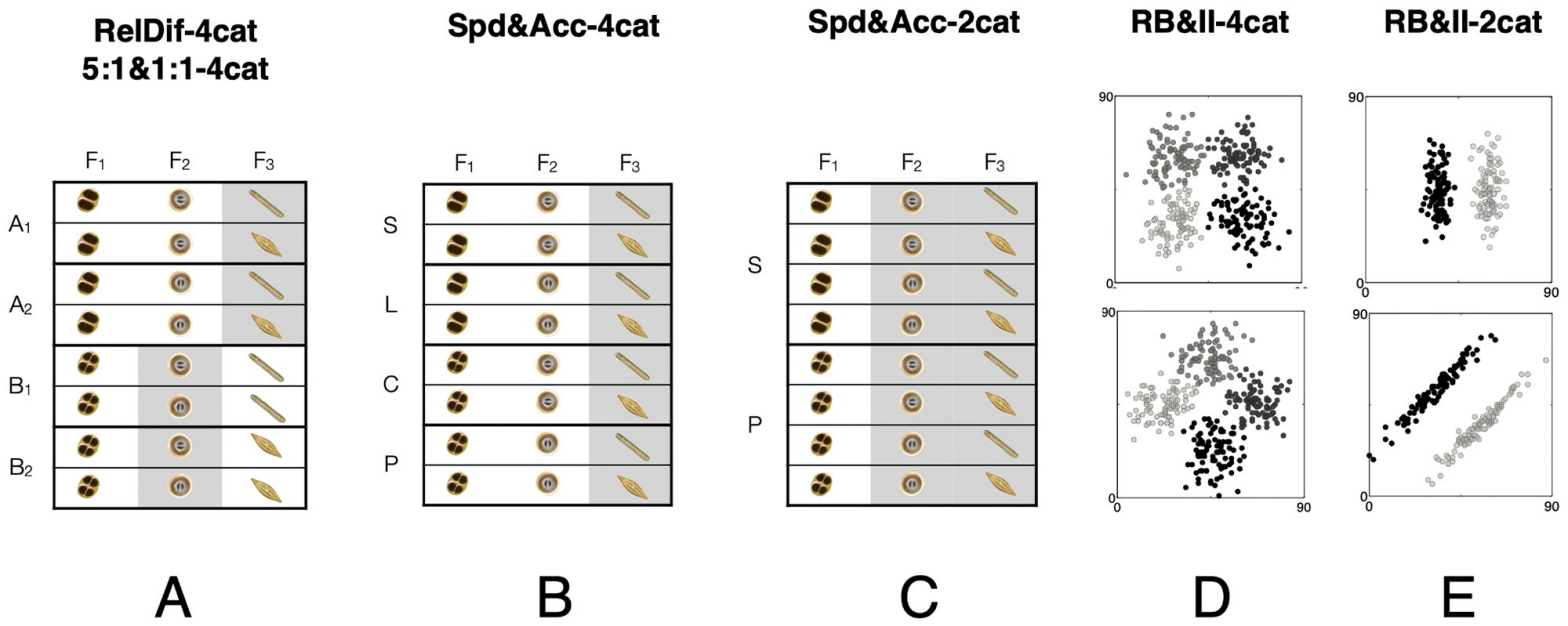
Category structures for the six experiments in order from L to R. Datasets 1 and 2 used the leftmost category structure (A). Datasets 3–6 used the remaining category structures (B-E). Features outlined in grey were irrelevant for categorization. Note that in Dataset 1, the two A categories have different relevant features than the two B categories. In Dataset 2, the irrelevant features were consistent for all categories. For Datasets 3 and 4, there was an additional manipulation of instructions to emphasize speed or accuracy. For Datasets 5 and 6, the rule-based categories are shown on top and the information-integration categories are shown on the bottom. Features were broadly similar to those used in Experiment 2, but were manipulated in Photoshop to have continuous values (size, curvature and rotation). There was an additional feature dimension not shown (thus, three in total) that was irrelevant for categorization.

*Stimuli and category structure*. Stimuli consisted of fictitious microorganisms with three binary-valued organelles, for a total of eight distinct stimuli to classify into four categories. The full stimulus subtended 16.3° of visual angle. Each feature was located centrally in each of the microorganism’s “arms”. Each feature subtended 1.3° and they were separated by 10.6° of visual angle. On every trial, exactly two of the organelles were informative as to category membership ("relevant" features), and one was not ("irrelevant" feature). Which two were relevant varied by trial—the value of one feature determined which of the other two was relevant. This category structure is illustrated in Fig 1A.

*Procedure*. Each trial began with a fixation cross. Gaze data from this portion were used to clean gaze information prior to analysis. The stimulus would then appear after participants clicked with a mouse.

**Fig 2:**
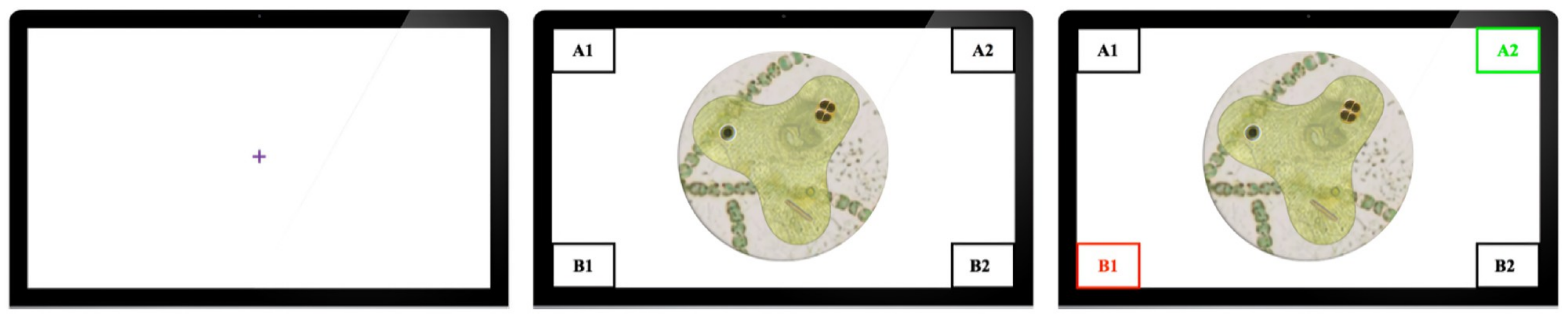
Phases of a trial. From left to right: fixation cross, response phase, feedback phase. The right panel shows the feedback button style used in all Datasets except Dataset 2 (5:1&1:1-4cat).

Participants made a category selection using the mouse, at which point the category buttons changed to provide feedback—if an incorrect response was made, the participant’s choice turned red and the correct answer turned green; otherwise, the response simply turned green. Participants could look at this feedback screen as long as they wanted (self-paced feedback) and moved on to the next trial by clicking on the mouse. See Fig 2 for an illustration of the phases of the trial.

The experiment consisted of a maximum of 272 trials, but could be completed early by those who learned. Participants reaching the learning criterion of 24 consecutive correct trials prior to trial #200 were only required to complete 72 trials after learning.

A Tobii X120 eye tracker sampling at 120 Hz was used to collect all gaze information. All subjects were seated 60 cm from the eye tracker.

#### Dataset 2: Base rate manipulation, relevance differences, 4 categories (5:1&1:1-4cat)

Dataset 2 was from a category learning experiment that investigated probability gain as a factor driving information access. The category structure was the same as in Dataset 1, but with an additional manipulation of the category base rates such that two categories were shown 5 times as often as the other two. Measures relevant to that manipulation were published in the second experiment of a paper by Meier and Blair [27]. The feedback processing data reported here were not investigated previously. This dataset is especially good for comparison with Dataset 1 as they share a category structure.

*Participants*. Participants were 116 undergraduates at Simon Fraser University recruited from introductory psychology classes. All had normal or corrected-to-normal vision. Of these, 23 were excluded from analysis for problems with gaze quality, and 6 more were excluded for responding randomly. This left a total of 87 participants included in analysis: 47 in the unequal frequency (5:1) condition, and 40 in the equal frequency (1:1) condition.

*Category structure and base rate manipulation*. Stimuli were similar to Dataset 1 and the category structure is shown in Fig 1A. Participants in the 1:1 condition saw all categories at an equal probability, while those in the 5:1 condition had two categories appear 5 times more often than the other two. For example, in a 24-trial block, participants would see ten A1 and A2 stimuli each, and two B1 and B2 stimuli each.

**Fig 3:**
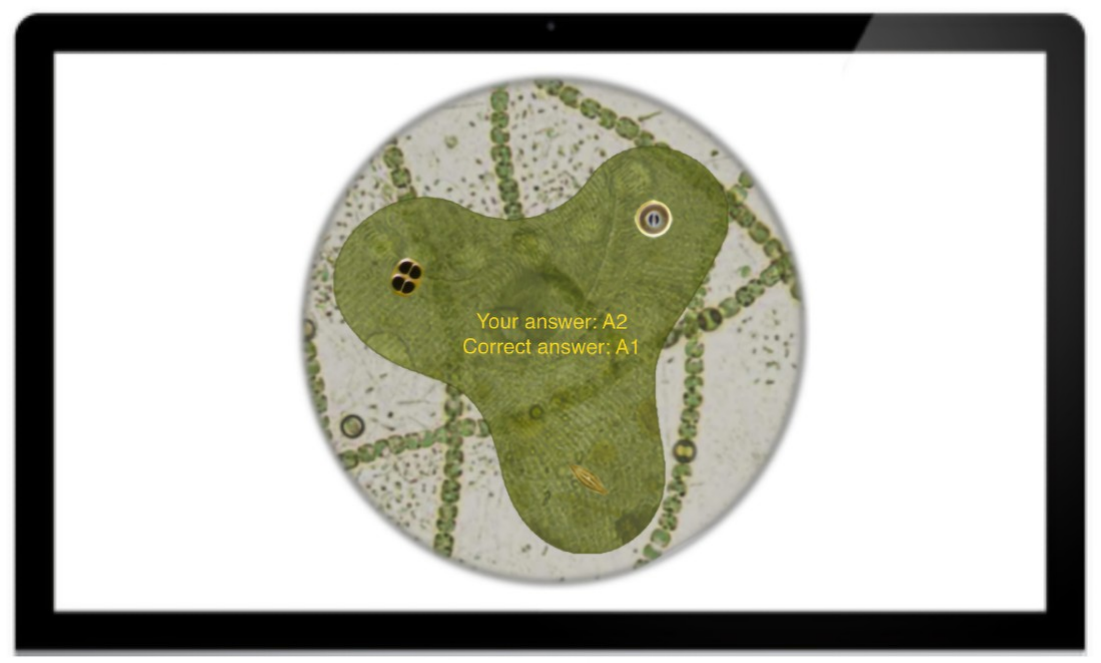
Dataset 2 (5:1&1:1-4cat), a gamepad was used to record inputs, and no response boxes were shown. **For** Feedback, which indicated the participants response and the correct response, was displayed in the centre of the screen as shown.

*Procedure*. Participants’ fixations were recorded with an eye tracker and responses were collected with a Logitech gamepad. Trials began with a centralized fixation cross. Participants pressed a button on the gamepad to reveal a stimulus. Participants made a category selection using the gamepad, at which point feedback was displayed as a 500 ms mask of green (correct) or red (incorrect). The stimulus was then re-presented along with the participants’ response and correct answer centrally displayed as shown in Fig 3.

#### Dataset 3: Speed accuracy, 4 category, with fixed feature relevance (Spd&Acc-4cat)

Dataset 3 was from a category learning experiment that investigated how instructions to emphasize speed or accuracy influence various attentional measures during category learning. The response phase data were reported by McColeman and colleagues as Dataset 2 [25], but the feedback phase data are analyzed here for the first time. This experiment used a less complex category structure wherein one of the three feature dimensions was always completely irrelevant. The feedback phase for this experiment was like Dataset 1 (RelDif-4cat).

*Participants*. Participants were 69 Simon Fraser University undergraduate students. 25 participants with bad gaze quality were dropped from analysis, leaving a total of 44: 20 in the speed-emphasis condition, and 24 in the accuracy-emphasis condition.

*Stimuli and category structure*. Stimuli were similar to previous datasets. The category structure was simpler than in the first two datasets, with the same two features relevant to every category, and the third feature consistently irrelevant. This is shown in Fig 1B. Apparatus, stimulus location, and feature visual angle remained the same.

*Procedure*. The experiment was grouped into speed-emphasis and accuracy-emphasis conditions, in which participants were instructed to prioritize either speed or accuracy in their responses. Each trial began with a fixation cross followed by presentation of the stimulus. Participants then chose a category using a Logitech gamepad. Feedback was presented similar to what is shown in Fig 3. Participants completed 300 trials in blocks of 20, with each block separated by a screen indicating their average response time and response accuracy.

#### Dataset 4: 2 category, with fixed feature relevance—Speed accuracy 1 relevant feature (Spd&Acc-2cat)

The experiment that produced Dataset 4 was nearly identical to that producing Dataset 3 (Spd&Acc-4cat). The only difference was this one had only two categories, so only one of the three feature dimensions was relevant, making for an easy-to-learn category structure. The response phase data were analyzed and presented by McColeman and colleagues [25] (Dataset 7), and the feedback data are presented for the first time here.

*Participants*. 69 undergraduate students from Simon Fraser University were recruited to participate in the experiment. 15 participants were excluded for bad gaze data, and one additional participant was excluded for random responding, leaving a total of 53 participants in the analysis: 29 in the speed-emphasis condition, and 24 in the accuracy-emphasis condition.

*Stimuli and category structure*. A two-category structure was used where only one feature determined stimulus category, as shown in Fig 1C. Stimuli were identical to the other speed & accuracy dataset (#3).

*Procedure*. Participants were first exposed to the fixation cross and stimulus, then made a selection using a Logitech gamepad. Feedback was given by re-presenting the stimulus and highlighting the correct response in green, and if the participant chose incorrectly, their incorrect choice was highlighted in red. Participants completed 300 trials in blocks of 20.

#### Dataset 5: 4 category rule-based and information-integration categories (RB&II-4cat)

Dataset 5 used stimuli with continuous-valued features rather that binary-valued features. Rule-based (RB) and information-integration (II) category structures are common (e.g., [28]), and are interesting for our purposes because there is some evidence from Smith et al. that there are feedback-related differences between RB and II categories [20].

Analysis of the response phase data was previously published [25], but the feedback processing data are new.

*Participants*. Participants were 78 Simon Fraser University undergraduates. 3 were unable to complete the experiment, 10 experienced equipment failure, 9 had poor gaze data, and 8 responded randomly. The remaining 48 participants were included for analysis: 25 in the information-integration condition, and 23 in the rule-based condition.

*Stimuli and category structure*. The experiment used a continuous rule-based or information-integration category structure with four categories as shown in Fig 1D. Stimulus features subtended 3° of visual angle and were spaced from each other by 10.6°. Though RB/II studies often use multi-dimensional stimulus features, the stimuli in this experiment had features separated into spatially distinct locations in order to facilitate eye tracking analysis of the data. Like the previously described stimuli, stimuli for this experiment had three distinct organelle-like features. However, the stimulus feature properties changed on a continuous scale to create the distribution of stimuli for a rule-based or information-integration category structure. These feature properties were rotation, size, and curvature.

*Procedure*. Subjects were tasked with categorizing 200 stimuli. Distance from stimulus to participant was measured to be approximately 70 cm. Each trial began with the subject using a mouse to click on a fixation cross to show the stimulus. Subjects would then click again to move into the categorization phase, at which point only the category labels were visible. Identical to previous experiments, once a category was chosen, the resulting feedback was shown; the stimulus was re-presented, and the correct category was highlighted in green, while an incorrect answer was highlighted in red.

#### Dataset 6: 2 category rule-based and information-integration categories (RB&II-2cat)

Our final dataset is similar to Dataset 5 (RB&II-4cat) except there were two categories instead of four.

*Participants*. 71 Simon Fraser University undergraduate students were recruited for this experiment. Data for 2 participants were excluded for random responding; the remaining 69 were included for analysis: 37 in the information-integration condition and 32 in the rule-based condition.

*Stimuli and category structure*. All stimulus features were identical to those used in Dataset 5. The category structure used is shown in Fig 1E.

*Procedure*. Experiment procedure was identical to Dataset 5 except that subjects categorized stimuli into two categories rather than four.

## Results

The primary aim of our analysis is to provide an accurate description of participant behaviour. More detailed research will be required to make causal claims about specific mechanisms, so we forgo complex followup analyses and only conduct the statistical analyses aimed at supporting the common sense reading of presented data. Each dataset was modelled separately. Many, but not all of the datasets include an experimental manipulation—for example Dataset 2 includes category frequency manipulation, and Dataset 3 includes a speed/accuracy manipulation. In all such cases, we include an effect for condition in the analysis. For all analyses, we used linear mixed effects models to test for main effects of relevant variables. The p-values for significance were determined by likelihood ratio tests of the full model with the effect in question present against the model with the effect absent. We discuss the significance of effects but do not lean on obtained model coefficient values for our conclusions. As such, we report these values in the Appendix rather than in the main text. All figures were made using the Gramm plotting library for MATLAB [29]. In all figures with separated panels, datasets 1–6 are presented with 1, 3, and 5 on the top row, and 2, 4, and 6 on the bottom, in that order.

Data were analyzed and visualized in bins of 24 trials each, with each bin representing the mean value of the trials it represents. This serves to make the graphs more legible and the data more normally distributed. In cases where a factor was excluded from the final model, graphs show results collapsed across that factor for the sake of clarity. Lastly, in the RelDif-4cat experiment (Dataset 1), participants were permitted to end the experiment early if they successfully met the learning criterion of 24 consecutive correct trials. As such, the final blocks have inordinately small numbers of participants, so we chose to omit the final two trial bins of data for this dataset to keep this variance consistent, leaving 10 trial bins for analysis.

### Question 1: How long do participants spend on feedback?

**Fig 4:**
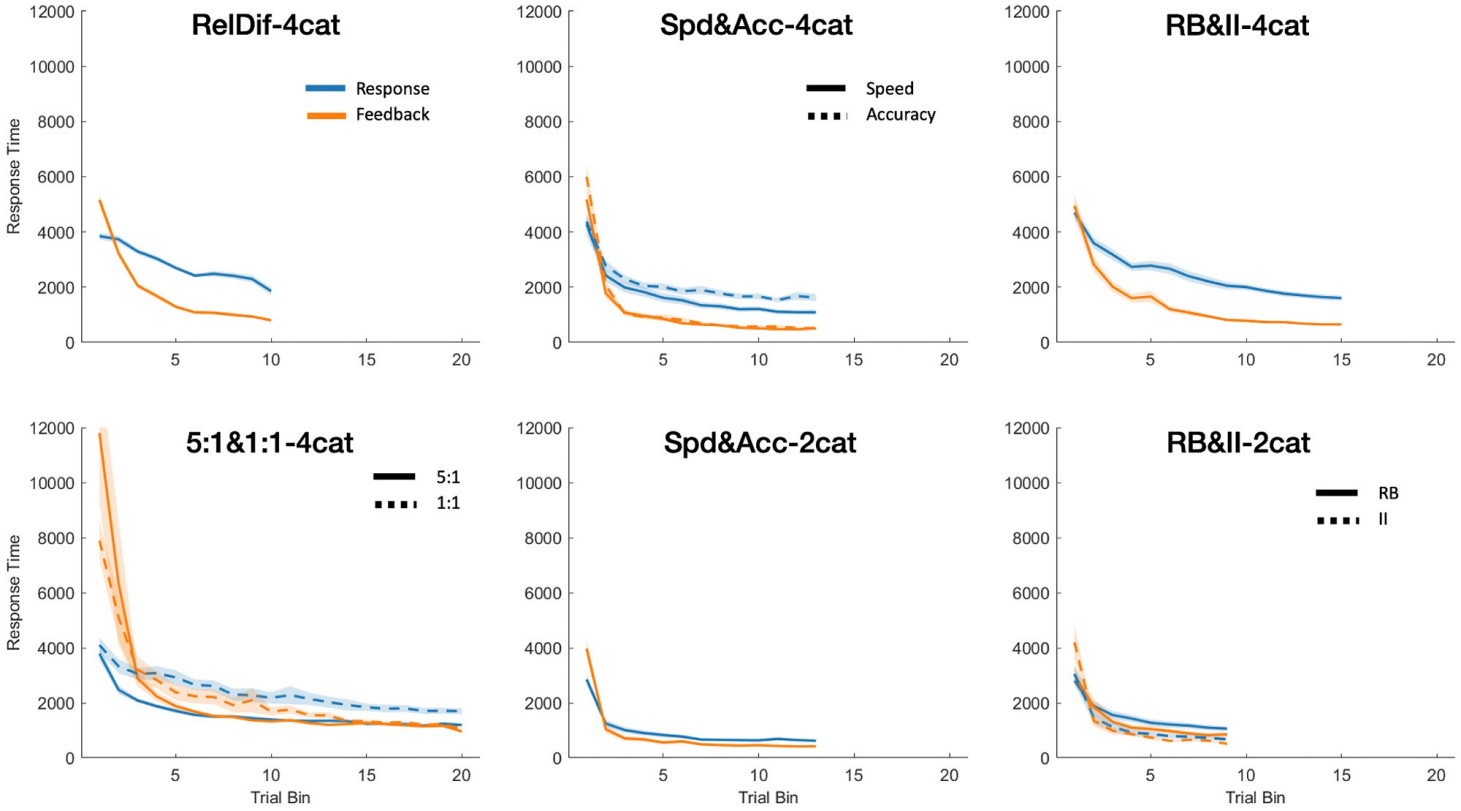
Response time during stimulus presentation and during feedback by trial bin. Each bin contains 24 trials and error shading represents SEM. In top-to-bottom order, the left-hand column contains Datasets 1 and 2, the middle column contains Datasets 3 and 4, and the right-hand column contains Datasets 5 and 6.

The experiments we analyzed all had self-paced feedback phases, so participants themselves chose how much time to spend reviewing the correct answer and the stimulus. The plots of response time for the response phase and the feedback phase for all datasets are shown in Fig 4. Participants showed a general decrease in time spent on both phases of the experiment as the experiment progressed. The maximum time spent was in the first block and there were fairly steep declines after that. For several of the datasets, participants spent more time on their responses than on the feedback, but this was not universally true.

To confirm these observations, response time was modelled as a function of trial phase (response or feedback), with experiment condition (included for any dataset with an experimental manipulation) and trial bin as fixed effects, and a random effect for intercept and trial grouped by subject. This was done separately for each dataset. We found that trial bin was a significant predictor of response time duration in all datasets (for Datasets 1–6, *χ*^*2*^(1) = 196.32; 51.11; 114.58; 57.23; 81.08; 66.10. All *p* <.001). The effects of trial phase varied per dataset; models from Datasets 1 (RelDif-4cat), 3 (Spd&Acc-4cat), 4 (Spd&Acc-2cat), and 5 (RB&II-4cat) estimated that feedback response times were shorter (*χ*^*2*^(1) = 384.31; 148.3; 5.85; 334.11, and *p* <.001; <.001; = .016; <.001, respectively), while the model from Dataset 2 (5:1&1:1-4cat) indicated the reverse (*χ*^*2*^ = 8.26; *p =* .004). Finally, Datasets 2 (5:1&1:1-4cat), 3 (Spd&Acc-4cat), and 6 (RB&II-2cat) had significant effects for condition (*χ*^*2*^(1) = 13.51; 10.51; 18.21, and *p* <.001; = .001; <.001, respectively) with the 1:1, accuracy and II conditions having the longer response times.

### Question 2: What do participants look at during the feedback phase?

**Fig 5:**
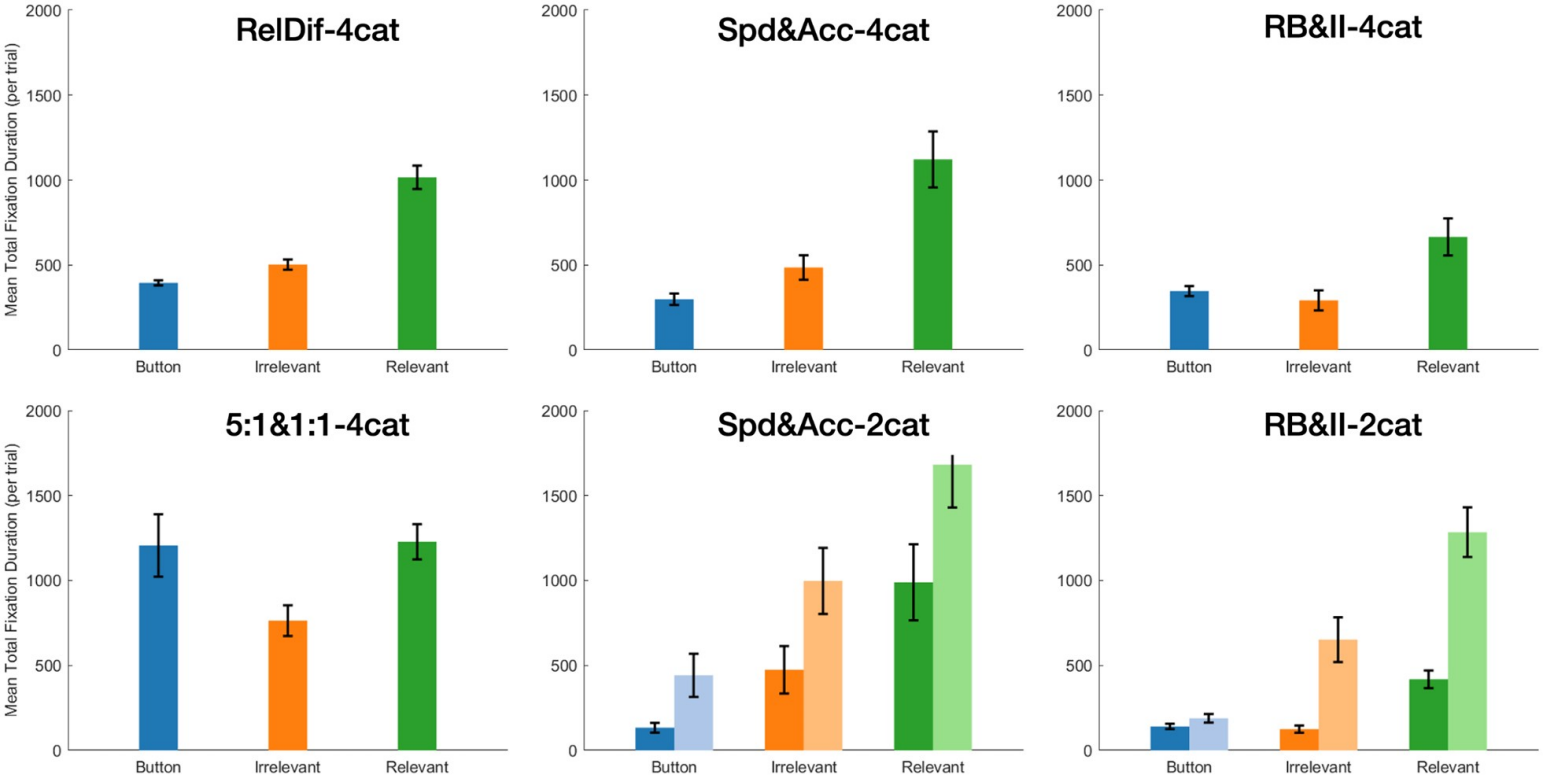
Mean time (per trial) spent fixating each area of interest (feedback button, irrelevant stimulus feature, relevant stimulus feature) for trials prior to reaching learning criterion. Conditions that were not significantly different were collapsed. Light bars represent the second condition (i.e., Accuracy, II). Error bars represent SEM. In top-to-bottom order, the left-hand column contains Datasets 1 and 2, the middle column contains Datasets 3 and 4, and the right-hand column contains Datasets 5 and 6.

Research on stimulus re-presentation suggests that participants can benefit from additional viewing of the stimulus following their response (e.g., [17]). We focused on three areas of interest: the relevant features of the re-presented stimulus, the irrelevant features of that stimulus, and the corrective feedback itself, which we call the feedback button. We disregarded fixations to other areas of the display. We also restricted the analysis to trials prior to the learning criterion of 24 consecutive correct responses. Fig 5 shows the total mean fixation duration per trial for the three areas of interest (AOI; relevant, irrelevant, button) for each dataset. Where condition was not a statistically significant factor, we collapsed those data for the purposes of plotting.

We find two features of these plots especially salient. First, participants spent significant time fixating stimulus features. In all datasets, the actual corrective feedback (i.e., button) was fixated less than half of the total time spent looking at these areas of interest. Given that the experiment phases were self-paced, and in our experience participants tend to be eager to finish, this represents a significant investment in time over the course of the experiment. These data echo findings that show that re-presenting the stimulus leads to improved performance in some cases [16]. Second, we see that in processing feedback, participants consistently emphasized relevant stimulus features over irrelevant ones. This was true even in the 2-category datasets, which had more irrelevant features than relevant ones. This suggests a role for selective attention even during feedback processing.

The importance of AOI and experimental condition on mean total fixation duration by trial was modelled using experiment condition (for datasets with experimental manipulations) and AOI as fixed effects with a random intercept for each subject. Experimental condition only proved significant for Datasets 4 (Spd&Acc-2cat) and 6 (RB&II-2cat) (*χ*^*2*^(1) = 6.23; 28.02 and *p* = .013; <.001, respectively). Area of interest, in contrast, was a significant contributor to the model in all datasets (*χ*^*2*^(1) = 272.09; 59.00; 107.14; 89.91; 111.09; 188.09. All *p* <.001).

### Question 3: Are there regularities in the order in which information is attended?

**Fig 6:**
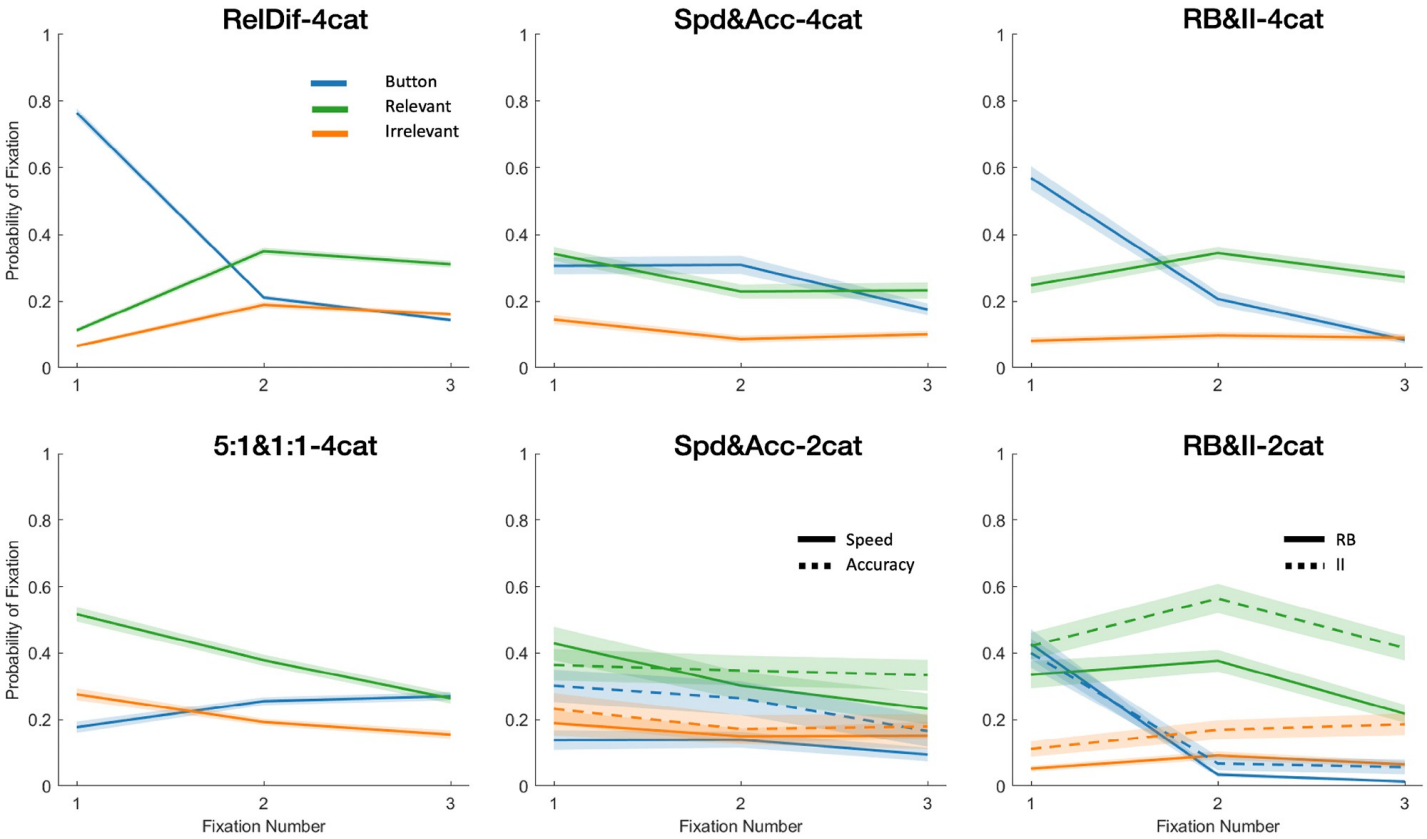
Probability of fixating each area of interest (feedback button, relevant stimulus feature, irrelevant stimulus feature) on each of the first three fixations of a trial. This is conditioned over all trials, whether or not they had three fixations. Conditions that were not significantly different were collapsed. Dashed lines represent the second condition (i.e., Accuracy, II). Shaded area represents SEM. In top-to-bottom order, the left-hand column contains Datasets 1 and 2, the middle column contains Datasets 3 and 4, and the right-hand column contains Datasets 5 and 6.

Our third analysis question investigates the order in which information was fixated during feedback. We again restricted analysis to data prior to meeting the learning criterion of 24 consecutive correct trials. In Fig 6 we show the probability of fixating each of the three areas of interest (relevant, irrelevant, button) for the first three fixations of each trial. Because not all trials have at least three fixations, the probabilities on fixations 2 and 3 are generally lower than on fixation 1, and the probabilities on each fixation do not always sum to 1. We note that experiments in which participants received feedback through the response buttons (RelDif-4cat (Dataset 1) and the two RB&II experiments (Datasets 5 and 6)) show high first fixations to buttons. The design of these particular experiments required participants to click on an onscreen feedback button with the mouse in order to begin the feedback phase of the trial, whereas the other experiments required pressing a button on a Logitech gamepad. The high proportion of first fixations to feedback was likely caused by participants keeping their gaze on the button after clicking on it directly. It also seems that relevant stimulus features were more likely to be fixated than irrelevant ones, echoing a similar pattern from above. There were significant differences in the conditions of Datasets 4 and 6 (Spd&Acc-2cat and RB&II-2cat, the two experiments with only two categories) with the accuracy and RB conditions more likely to be fixated. This also echoes the finding from Question 2 above; as total fixation duration and probability of fixating are likely correlated measures, this is understandable. We note that fixation probabilities for all areas of interest go down towards the end of the experiment. This happens because participants make less fixations during feedback as they learn; the later in the experiment they get, the less likely there is a second or third feedback fixation at all. We find no other theoretically interesting effects in the plots, and no evidence of interesting participant strategies.

We modelled the probability of fixation as a function of location (relevant feature, irrelevant feature, feedback button), fixation order (first, second, or third of the current trial), and experimental condition (for datasets with experimental manipulations), with random intercepts for each subject. For all datasets, fixation order proved a significant predictor of fixation probability, meaning that there were significant differences between the first, second, and third feedback-phase fixations of each trial. For Datasets 1–6, *χ*^*2*^(1) = 305.82; 220.78; 152.5; 50.95; 288.67; 190.92, respectively. All *p* <.001. Likewise, AOI was also a significant predictor in all datasets, *χ*^*2*^(1) = 2210.9; 354.13; 223.45; 186.14; 968.49; 441.58. All *p* <.001. Condition was significant only for Datasets 4 (Spd&Acc-2cat) and 6 (RB&II-2cat), *χ*^*2*^(1) = 5.90; 13.92, respectively; *p =* .*015*, <.001.

### Question 4: Do participants use a different allocation of attention during the response and feedback phases?

Our fourth research question asks how the allocation of attention during the response phase compares to the allocation during the feedback phase. One possibility is that participants use the feedback phase to look at any information they did not look at during the response phase, leading to complementary allocations of attention. Another possibility is that participants employ increasingly consistent and relevance-focused attentional allocations, leading to converging allocations of attention between response and feedback phases. There is some support for this second idea [22].

**Fig 7:**
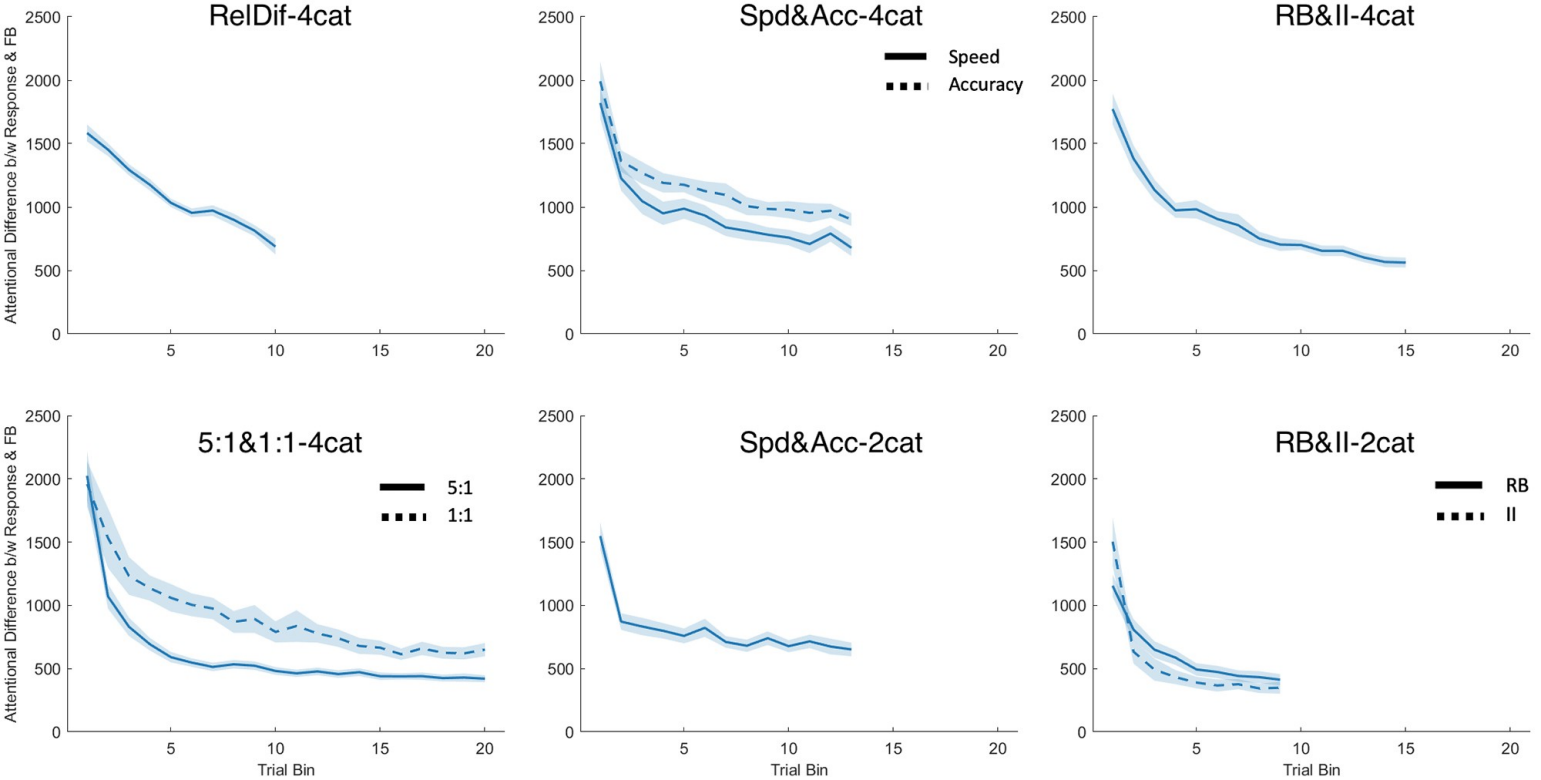
Differences in the allocation of attention between the response and feedback phase plotted over trial bins by dataset and condition. Shaded areas represent the SEM. In top-to-bottom order, the left-hand column contains Datasets 1 and 2, the middle column contains Datasets 3 and 4, and the right-hand column contains Datasets 5 and 6.

To determine the similarity between response and feedback-phase attention allocation, we represented attention to the stimulus in each phase as a point in 3D space (one dimension for each stimulus feature—see Fig 1), with attention defined as the total amount of time spent fixating that feature. The final value was calculated as the Euclidean distance between the points in the response phase and feedback phase of the same trial. For example, if a participant looked at Feature 1 for 1000ms, Feature 2 for 1000ms and Feature 3 for 0ms during the response phase, but Feature 1 for 0ms, Feature 2 for 0ms and Feature 3 for 1000ms, then the euclidian difference would be 1732 ms. On the other hand, a value of 0 would mean attention to stimulus features was identical between the response and feedback phases. The obtained differences in attentional allocation are shown in Fig 7.

We modelled attentional distance by experimental condition (for datasets with experimental manipulations) and trial bin, with random slopes for trial bin grouped by subject. Trial bin was a significant predictor for all datasets: *χ*^*2*^(1) = 113.06; 79.00; 61.26; 27.48; 68.99; 65.95, with all effects having *p* <.001. Experimental condition was a significant predictor in Datasets 2 (5:1&1:1-4cat), 3 (Spd&Acc-4cat), and 6 (RB&II-2cat), *χ*^*2*^(1) = 15.47; 6.93; 4.53 respectively. Effects had *p* <.001; = .008; = .033, respectively.

To account for the possibility that the convergence shown might be the result of decreasing overall time spent during both phases of the experiment, we performed two additional analyses to ensure we could present a clear picture of the participants’ behaviours. First, we investigated the possibility that participants might trade off their time in the response phase with time in the feedback phase such that some participants spend the bulk of their time during the response phase, while others spend theirs during feedback. Total time spent on feedback was subtracted from total time spent pre-response on a trial by trial basis. We examined the distributions of these difference values by experiment, and found they were unimodal and appeared normal. They showed the incidence of participants taking a short time during response and long time during feedback (indicating a trade-off strategy) was exceedingly rare.

**Fig 8:**
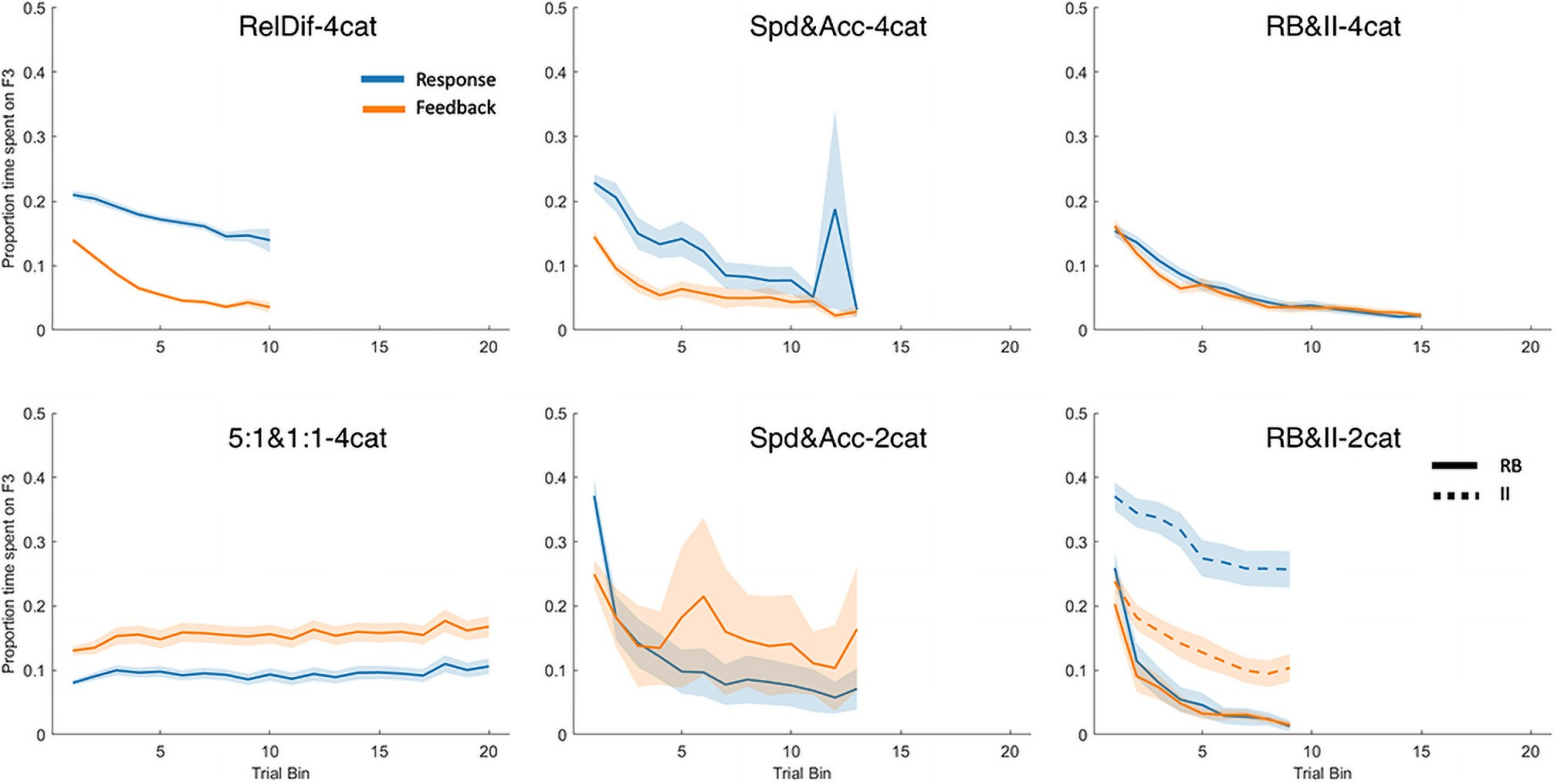
Differences in the proportion of attention allocated to the irrelevant feature between the response and feedback phase plotted over trial bins by dataset and condition. Shaded areas represent the SEM. In top-to-bottom order, the left-hand column contains Datasets 1 and 2, the middle column contains Datasets 3 and 4, and the right-hand column contains Datasets 5 and 6.

Our second analysis sought to confirm that participants looked at the same information during response and feedback, and rule out decreasing durations as the primary cause of convergence. Previous results show that participants learn to focus their attention on relevant information, gradually reducing the time spend on irrelevant information as the experiment progresses [25]. If participants do the same during feedback, we can be sure that the convergence we find is not only in decreased time overall, but in a similar allocation of attention to relevant stimulus features. We calculated the proportion of time spent on irrelevant stimulus features during the feedback phase and pre-response, and took the difference on a trial by trial basis. This should remove any effect of decreases in total time from our results. As shown in Fig 8, we found that in most cases the difference in proportion time spent on irrelevant features between the two trial phases decreased. The exceptions were Datasets 1 & 2, in which the difference increased slightly. These two datasets employ the more complex category structure where all features are relevant at least some of the time, and so perhaps Leong et al.’s finding of converging attention [22] only applies to simpler tasks. Statistically, trial bin was a significant predictor for all datasets except Dataset 4 (Spd&Acc-2cat) (*χ*^*2*^(1) = 27.71; 8.45; 13.73; 43.8; 23.15 for Datasets 1–3, 5, and 6 respectively, with all effects having *p* <.001 except Dataset 2 where p = .004). Condition was only a significant factor in Dataset 6, where RB and II conditions were different (*χ*^*2*^(1) = 33.01, p <.001).

### Question 5: What are the characteristics of individual fixations in the response and feedback phases?

There is some evidence that the properties of individual gaze fixations during category learning can vary based on learning [26]. Our fifth analysis question asks if there are any of these kinds of effects during feedback processing. We restricted our analysis to trials prior to the learning criterion of 24 consecutive correct responses. Average durations for fixations were within the 200-400ms range, except for Datasets 3 and 4 which were a bit higher. The effects on the duration of individual fixations were generally small and somewhat inconsistent, and so we do not include a figure. We modelled mean fixation duration as a function of trial phase (response or feedback), with additional fixed effects for area of interest (relevant stimulus feature, irrelevant stimulus feature, feedback button) and trial bin, and random slopes for trial bin grouped by subject. Trial bin was a significant predictor in all datasets except Dataset 3 (Spd&Acc-4cat); Datasets 1, 2, 4–6, *χ*^*2*^(1) = 125.08; 57.55; 9.44; 47.56; 57.20 with *p* <.001 except for Dataset 4 (Spd&Acc-2cat) where *p =* .002. Fixations generally shrunk as the experiment progressed, except for Dataset 4 (Spd&Acc-2cat), where they lengthened. Trial phase was a significant predictor in all datasets. Datasets 1–6, *χ*^*2*^(1) = 588.98; 367.78; 8.55; 15.93; 37.43; 61.74. All *p* <.001, except for Dataset 3 (Spd&Acc-4cat) which had *p =* .003. In Dataset 2 (5:1&1:1-4cat), feedback phase fixations were longer than response phase fixations. In all other datasets, response phase fixations were slightly longer. Area of interest was significant across all datasets, with fixations to relevant stimulus features longer than fixations to feedback buttons, and sometimes longer than fixations to irrelevant stimulus features: for Datasets 1–6, *χ*^*2*^(1) = 345.83; 300.95; 109.15; 56.78; 846.24; 67.54. All *p* <.001.

Overall, the durations of individual fixations seemed to be influenced by various factors at play in feedback processing. We saw shorter fixations during feedback and when processing the feedback signal (as opposed to the stimulus). Fixations also shrank in most cases as the experiment progressed. We hasten to note that these effects are small, with differences in the low tens of milliseconds, not hundreds of milliseconds. As such, these findings may be of interest to researchers working on saccadic timings and oculomotor control, but may not rise the level of important for researchers focused on learning.

### Question 6: Do participants inspect feedback differently after they make an error?

Given the relationship between attention and learning, one could expect that participants would treat the feedback phase on an error trial differently than they would following a correct response (e.g., [17]).

**Fig 9:**
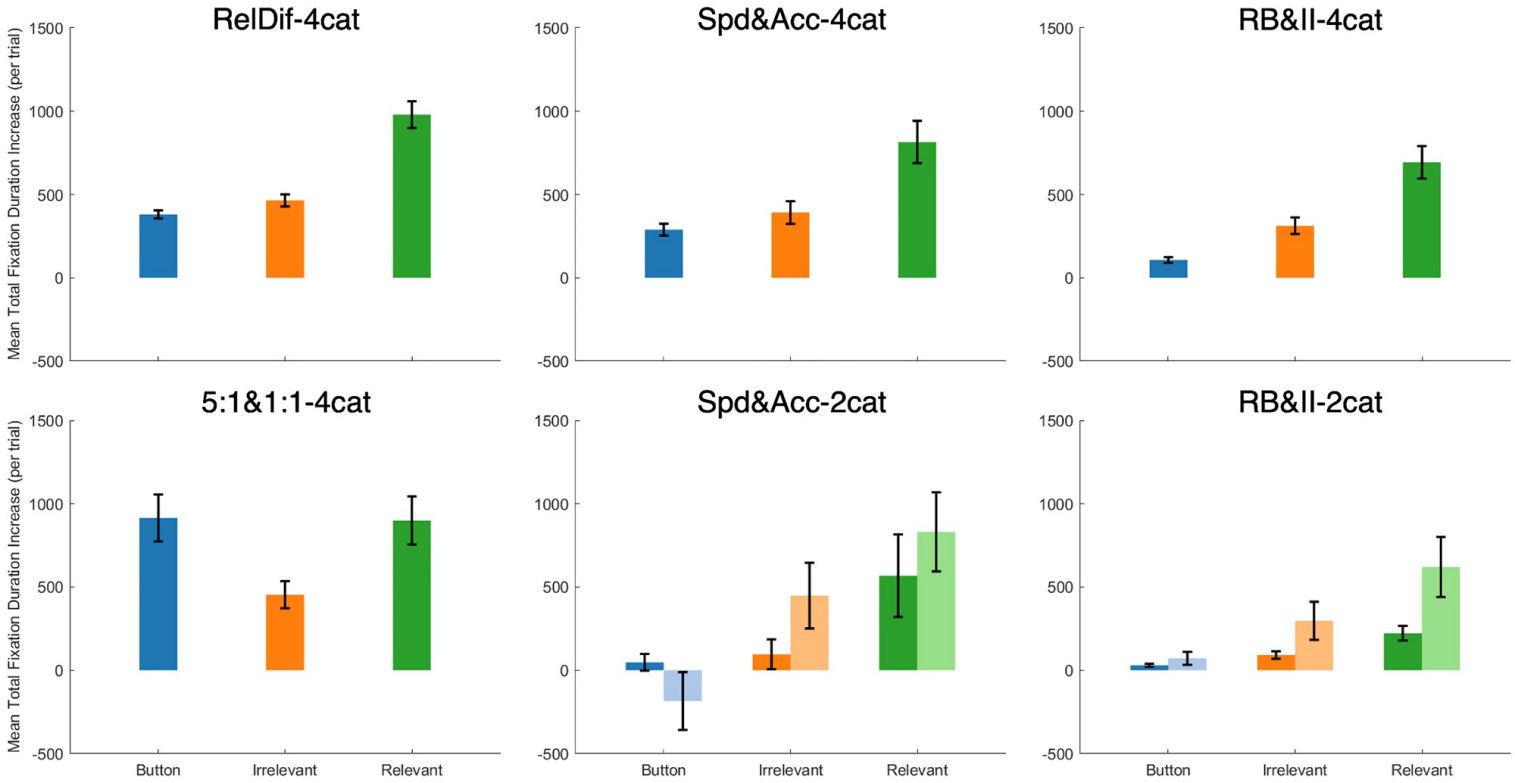
Mean difference (error trials minus correct trials) in total time fixating each AOI (feedback button, relevant stimulus feature, irrelevant stimulus feature), plotted by dataset and condition. Conditions that were not significantly different were collapsed. Lighter bars represent the second condition (i.e., Accuracy, II). Error bars represent SEM. In top-to-bottom order, the left-hand column contains Datasets 1 and 2, the middle column contains Datasets 3 and 4, and the right-hand column contains Datasets 5 and 6.

Fig 9 shows the difference between total fixation durations per trial on incorrect trials and correct trials. First, we note that in almost every case, values were positive, indicating that participants spent more time on feedback after a mistake. Second, the overall patterns were similar to Fig 5, indicating that the extra time spent on incorrect trials was just more of the same—emphasizing the stimulus and relevant features in particular—rather than an altogether different approach.

Statistically, we modelled mean total fixation time per trial as a function of area of interest (relevant, irrelevant, button) and trial accuracy (correct, incorrect), as well as experimental condition (for datasets with experimental manipulations), with random intercepts for each subject. We found that including trial accuracy significantly improved model fit for all datasets (*χ*^*2*^(1) = 404.92; 161.32; 32.67; 19.62; 60.52; 24.61. All *p* <.001.) We also found that area of interest significantly improved model performance. Datasets 1–6 (*χ*^*2*^(1) = 497.38; 111.94; 199.35; 149.08; 235.38; 316.80. All *p* <.001). Only Dataset 4 (Spd&Acc-2cat) and 6 (RB&II-2cat) showed improvement by including condition: *χ*^*2*^(1) = 5.97; 27.53 with *p =* .015; <.001, respectively.

One possibility is that errors are longer simply because they tend to occur earlier in the experiment, when feedback phases are longer overall. To check that these findings were not simply the result of there being less error trials as learning increased, we did the same analysis using only the last 10 trials prior to learning criterion being reached. We found a similar pattern of increased time spent on feedback, and on all areas of interest, on error trials than correct trials, even when limiting the range of trials included.

### Question 7: What happens when people ignore feedback altogether?

**Fig 10:**
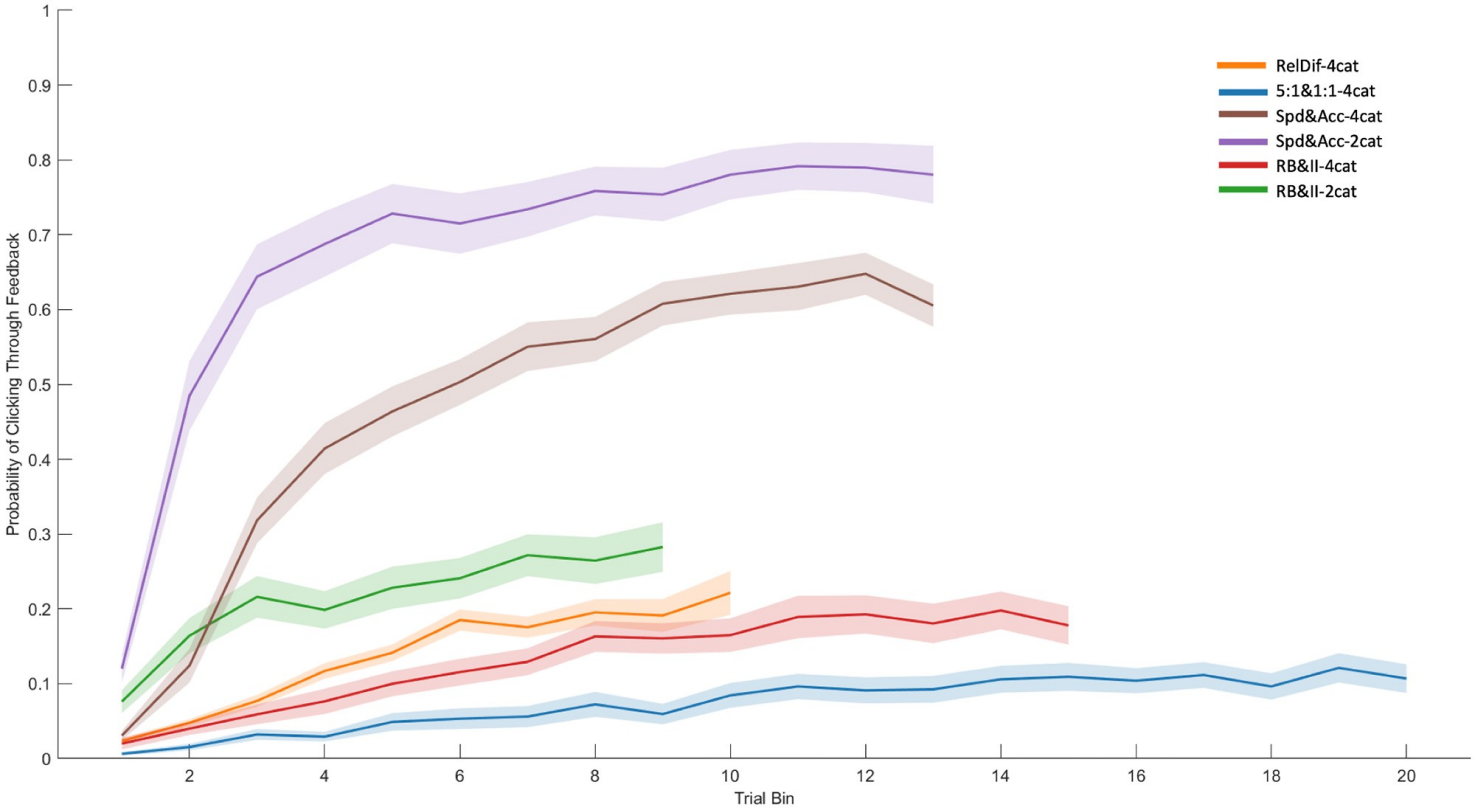
The probability of clicking through the feedback phase with no fixations, plotted by trial bins and dataset. Shaded area represents SEM. In top-to-bottom order, the left-hand column contains Datasets 1 and 2, the middle column contains Datasets 3 and 4, and the right-hand column contains Datasets 5 and 6.

Our seventh and final analysis asks how often people choose to simply skip the feedback altogether. This is a phenomenon currently unaccounted for by models of category learning, which assign a feedback signal boosting connection weights on every trial regardless of whether a human participant would have even looked at the feedback or not. We define feedback clickthroughs as trials where participants made no informative fixations—that is, no fixations to stimulus features or feedback buttons. Fig 10 plots the prevalence of clickthroughs by trial bin. In all datasets, the probability of clicking through feedback increased as the experiment went on—the more the participant had learned, the less they appeared to need to look at the feedback signal or the re-presented stimulus. The magnitude of change varied greatly by experiment, but the phenomenon appeared to some extent in all datasets, increasingly so in later trials.

We modelled the probability of clickthrough by trial bin with random slopes for trial bin grouped by subject. Trial bin was significant for all datasets (*χ*^*2*^(1) = 142.07; 32.09; 99.94; 58.01; 32.52; 35.88. All *p* <.001). Condition (included for datasets with experimental manipulations) was not significant in any dataset.

It seems as though prevalence of clickthroughs is related to the difficulty of the task, with easier category structures having more clickthroughs. We note that, though many clickthroughs are very fast, there is the possibility that participants are still able to covertly attend some information, a possibility also noted by Meier and Blair [27]. Understanding covert attention in the context of information access seems like a worthwhile avenue for future research.

## General discussion

Much of the recent research on learning from feedback is focused on specific effects, including delaying feedback information (e.g., [30]), probabilistic/contingency-based models (e.g., [31]), and phrasing of feedback (e.g., as positive or negative [32]). In contrast, the primary goal of the present work was to build a basic understanding of how people process feedback in a category learning experiment. Feedback is inarguably important for learning, yet the dominant models within category learning treat it as a simple teaching signal that is automatically processed and stored—something useful in the learning process, but lacking nuance Here, we investigated participants’ gaze data during feedback processing in datasets from six category learning experiments (each with varying category structures and manipulations), a total of 505 participants, in order to support or reject this simplified view. Our findings demonstrate that the simple view of feedback overlooks participant behaviour in three important ways.

First, the simplified view overlooks the clear importance of stimulus re-presentation, which is the dominant target of gaze by participants, garnering more attention than the feedback itself. Further, when participants spend more time on feedback after mistakes, they spend it mostly looking at the features of the re-presented stimulus. This was true regardless of category structure or difficulty of learning. The prominence of the stimulus as a fixation target may be related to the complexity of the stimulus. The categories were alphanumeric and thus were very familiar to participants; the stimuli, in contrast, were novel and relatively complex microorganisms. Future research might swap the features for the categories so that the features are alphanumeric and the categories are the novel and complex visual features to investigate this possibility. Regardless of the outcome of such an experiment, our findings replicate the prominence of stimulus viewing during feedback of Watson and Blair [17], and are consistent with findings like those of Bourne and colleagues and Halff that show that re-presenting the stimulus provides important benefits while learning categories [16, 33].

Second, because the simple view treats feedback on correct and error trials as identical, it also fails to account for the fact that participants change their behaviour if they make a mistake. Feedback processing differs between correct and error trials: participants spend more time on the feedback phase when they make errors. This finding has two different aspects. First, the idea that additional study time, and thus additional co-activation of features and category labels, will improve memory is not new (e.g., [34]). Existing category learning models, though, are not constructed to address temporal manipulations post-response at all; they are built around the idea of one incremental change per trial. Second, there is no self-monitoring mechanism in these models to allow the flexibility to spend additional resources to improve learning on specific trials. The flexibility shown here by participants is reminiscent of how participants can selectively attend to features depending on the value of other features [11], and argues for adding these mechanisms to extant models.

The third major finding is that later in the experiment, participants increasingly choose to skip the processing of feedback altogether, clicking on to the next trial without fixating any relevant information. This happens rarely at the beginning of the experiment, but increases in prevalence as the experiment progresses. Of all the present findings, this effect seems most influenced by category structure: the most difficult categories have small numbers of clickthroughs, but the easier categories have very high clickthrough rates. This makes sense, though we note that existing models assume the stimulus exemplar is processed and stored on each trial, regardless of accuracy or need, which appears not to reflect the actual behaviour of human participants.

There are also a few curios within these analyses. Dataset 2 in particular stood out in several key ways. The time spent on feedback was extraordinarily long, especially in the 5:1 condition (Fig 4) during the first few blocks of the experiment. Further, the additional time spent during feedback seems to be allocated to the feedback button more for this dataset than any other dataset (Fig 7), and the probability of clicking through feedback is least of all the datasets. We would guess that the explanation involves at least a combination of two factors. First, it is the most complex category structure, wherein the features are relevant are different for different categories. McColeman et al., reported a large number of fixations to irrelevant features compared to simpler categories and slower learning [25]. The second factor, and one that separates it from Dataset 1 is that the feedback buttons are central, rather than in the corners. We expect this combination encouraged more inspection of feedback buttons than in datasets with only one of those factors. More focused research will be needed to understand how feedback placement and task difficulty might influence feedback processing.

The durations of individual fixations, which might have been longer during feedback, reflecting more careful encoding of stimulus features, are actually shorter than response phase fixations in all datasets except RelDif-4cat, with all differences quite small in magnitude. Fixations to relevant stimulus features were consistently longer than fixations to feedback signals, suggesting more careful attention to the stimulus than the feedback signal when processing feedback.

The fixation order analysis, which sought to reveal if participants might be strategically investigating stimulus features and feedback in a particular order, mostly shows that participants varied across fixations, but not substantially. The exception to this is that in those experiments where participants selected their category choice with a mouse (Datasets 1, 5, and 6), they tended to look at the category feedback first. Given that their attention was already on the feedback when the feedback phase began, this makes sense, but does not seem to have important implications for theory. Relevant features maintained a higher likelihood to be fixated than irrelevant features across the first three fixations of all datasets.

At the broadest level, our three main findings reveal that selective attention is ubiquitous throughout the whole category learning task. It functions to emphasize the importance of certain stimulus features, the helpfulness of extra stimulus encoding during times of uncertainty, and the superfluousness of feedback once one has mastered the task. This ubiquity serves as a reminder that there is value in thinking about, and implementing, the basic principles guiding cognitive processing as generally as possible. Extant models of category learning allow for selective attention only to stimulus features for the purpose of improving category learning decisions (e.g., [8]), and indeed, it is an established finding that participants weigh the importance of information when choosing to access it [11, 12]. But, our study shows clearly that this restriction is unnecessary and inaccurate and that human cognition is dynamic and flexible. Gottlieb argues that too often researchers study decision-making independently from the active sensing behaviours with which they are coordinated [23], and that seems to be the case in the current situation. Theoretical work that aims at understanding active sampling [23] conjoined with decision processes seem better suited to characterizing our findings than more specific models despite those models’ focus on our exact experimental paradigm.

The dynamical field model of learning, attention and gaze, LAG-1, is an example of an integrated approach [24]. Without fitting the model explicitly, it is impossible to make quantitative assertions about the model’s ability to account for every aspect of these particular data, but most of our findings seem compatible with the theoretical framework outlined by LAG-1. The model naturally predicts improved response times during response and feedback phases because learning boosts activations and thus reaching thresholds faster. It also predicts the present finding that people prefer relevant features to irrelevant ones in both response and feedback phase. Though LAG-1 predicts convergence—learning drives attention toward all and only the relevant features in both phases of the experiment, it is unclear to us whether or not LAG-1 could account for the degree of attentional convergence found here. Early in learning, before LAG-1 has much information about which features indicate which categories, LAG-1 will tend to fixate all three available features before making a choice, keeping attentional allocations somewhat similar. Humans, however, will often fixate only one or two features before deciding (perhaps in an attempt to find a simple rule that correct classifies the stimuli). To the degree that the magnitude of our convergence measure depends on that variability, it may be out of reach for LAG-1. The difference between correct and incorrect trials is another example where LAG-1 predicts the correct qualitative pattern. In the model, these differences arise from competitive dynamics between the category nodes, which delay the response to move to the next trial. During that delay, the attentional system will prioritize informative features, just like during the response phase, producing findings similar to the present findings. Finally, other modelling efforts with LAG-1 (currently under review) have demonstrated the increasing likelihood of skipping feedback altogether. This occurs because the threshold to begin the next trial can be initiated immediately if the category activation is strong enough. Two of the findings from Dataset 2 seem likely to be difficult for the model to capture. The findings that attention diverges rather than converges, and that fixation durations are longer during feedback than during the response phase may be due to some strategic mechanisms engaged by the difficulty of the task that are not currently part of LAG-1.

Not all research will want or need to simulate the full scope of dynamic human information access in category learning tasks as LAG-1 does. Still, researchers using models that simplify feedback processing can benefit from our findings without the added complexity of creating a fully dynamic model in a couple of ways. First, participants in the present experiments clearly prioritize looking at the re-presented stimulus, and researchers running category learning experiments should remember that participants consider this information worth their time and make it available to them. Second, most models use selective attention for category decisions, but none that we know of use it for feedback processing. Applying the same set of attention weights to the learning processes, for instance by storing weighted features of category exemplars, seems a relatively simple inclusion that will bring models more in line with the human behaviour we see in the diverse categories we reviewed here.

Differences in processing feedback when learning RB and II categories have been of interest [28]. According to Smith and colleagues’ study, a rule-based structure is learned through iterative testing of hypotheses, requiring the use of working memory, while information-integration structures are learned through reinforcement signals that are sensitive to temporal delays [20]. They found that deferred feedback significantly impaired the performance of subjects learning implicit structures because of these underlying differences. However, Le Pelley and colleagues argued that this effect was confounded with task difficulty and expanded on this study by adding another category structure; a conjunctive rule-based structure that operated on two-dimensions [35]. It was found that manipulating feedback showed no effect on category structure when comparing between the conjunction and implicit structure, while significant interactions were found between the unidimensional rule-based structure and the conjunction structure. This supported Le Pelley and colleagues’ view that deferred feedback dissociated between difficulty, and not the underlying mechanisms in learning category structures. In a similar fashion, we have found that manipulating between rule-based and information-integration structure conditions showed significant differences for the majority of our measures in Dataset 6 (2-cat), but not Dataset 5 (4-cat). Given that these two datasets differ only in the number of categories used, it seems that difficulty, not category structure, may be responsible for changes in how we process feedback, as proposed by Le Pelley and colleagues [35].

In addition to studying feedback to understand category representation, there is also a theoretical interest in learning algorithms that drive change in learning and attention shifts. While not all models use supervised methods (e.g., [7, 24]) some models rely on error-driven learning algorithms (e.g., [8, 9]). The evidence for error driven learning of attention is mixed, and seems to rule out a simple universal error-driven mechanism for both learning and attention. For example, McColeman et al. tracked attentional change across trials of a wide variety of category learning studies segregating correct and incorrect trials [25]. They found that for many (but not all) datasets, there was no bias toward attentional change during error trials. A study by Blair, Watson & Meier showed that, in the category structure they used, attention shifts occurred largely after participants learning the correct classifications, rather than concurrently [11]. They also found that attentional optimization continued long after participants stopped making mistakes. Further, in the experiment, they stopped receiving any category feedback at all after they met a learning criterion of 24 consecutive correct trials. Despite receiving no feedback at all, attention continued to shift away from irrelevant stimulus features. On the other hand, work by Don and colleagues notable especially because they examined attention during feedback, found patterns consistent with error driven learning [14]. Further research is needed, clearly, but we are confident that a complete account of the influence of error on attention and learning will include data from all phases of the category learning task.

The datasets analyzed here were from experiments not explicitly designed for studying feedback processing. That is an advantage in that they reveal feedback processing under normal, un-manipulated conditions that might occur in a typical experiment, and allow for a baseline comparison for future research. The downside, however, is that without a direct manipulation of feedback presentation, we cannot make precise causal claims that one might be able to make if one, for example, manipulated the duration of the feedback phase to investigate its causal impact on learning. We thus see our work as part of the earliest steps of research that taps into an unmined vein of research possibilities that involve memory, attention, and categorization in the category learning task as a whole.

All the feedback phase data used in the analyses are available at http://summit.sfu.ca/item/21376. Data for the response phase of the published experiments are currently available at http://summit.sfu.ca/item/12716 (Dataset 1), http://summit.sfu.ca/item/12715 (Dataset 2), http://summit.sfu.ca/item/12719 (Datasets 3 and 4), http://summit.sfu.ca/item/11827 (Dataset 5), http://summit.sfu.ca/item/12718 (Dataset 6). These experiments were not preregistered.

We would like to thank current members of the Cognitive Science Lab. We would also like to thank past members of the lab, especially Kim Meier and Lihan Chen for significant help in collecting these data.

## Supporting information

S1 Table Details of LME models. Includes results of best fitting LME models for all 6 research questions, including analysis 4.1 (additional under research question 4).
(PDF)

## References

[1] Hull CL. Quantitative aspects of evolution of concepts: An experimental study. Psychol Monogr 1920 28(1): i–86. 10.1037/h0093130

[2] Rosch E, Mervis CB. Family resemblances: Studies in the internal structure of categories. Cogn Psychol 1975 7(4): 573–605. 10.1016/0010-0285(75)90024-9

[3] Smith JD, Minda JP. Thirty categorization results in search of a model. J Exp Psychol Learn Mem Cogn 2000 26(1): 3–27. 10.1037//0278-7393.26.1.3 10682288

[4] Blair MR, Homa D.As easy to memorize as they are to classify: The 5–4 categories and the category advantage. Mem Cognit 2003 31(8): 1293–1301. 10.3758/BF03195812 15058690

[5] Medin DL, Schaffer MM. Context theory of classification learning. Psychol Rev 1978 85(3) 207–238. 10.1037/0033-295X.85.3.207

[6] Nosofsky RM. Attention, similarity, and the identification-categorization relationship. J Exp Psychol Gen 1986 115(1): 39–61. https://pubmed.ncbi.nlm.nih.gov/2937873/ 2937873 10.1037//0096-3445.115.1.39

[7] Love BC, Medin DL, Gureckis TM. SUSTAIN: A network model of category learning. Psychol Rev 2004 111(2): 309–332. 10.1037/0033-295X.111.2.309 15065912

[8] Kruschke JK. ALCOVE: An exemplar-based connectionist model of category learning. Psychol Rev 1992 99(1): 22–44. http://www.ncbi.nlm.nih.gov/pubmed/1546117 1546117 10.1037/0033-295x.99.1.22

[9] Kruschke JK, Johansen MK. A model of probabilistic category learning. J Exp Psychol Learn Mem Cogn 1999 25(5): 1083–1119. 10.1037//0278-7393.25.5.1083 10505339

[10] Lamberts K. The time course of categorization. J Exp Psychol Learn Mem Cogn 1998 24(3): 695–711. 10.1037/0278-7393.24.3.695

[11] Blair MR, Watson MR, Meier KM. Errors, efficiency, and the interplay between attention and category learning. Cognition 2009 112(2): 330–336. 10.1016/j.cognition.2009.04.008 19481733

[12] Rehder B, Hoffman AB. Eyetracking and selective attention in category learning. Cogn Psychol 2005 51(1): 1–41. 10.1016/j.cogpsych.2004.11.001 16039934

[13] Matsuka T, Corter JE. Observed attention allocation processes in category learning. Q J Exp Psychol 2008 61(7): 1067–1097. 10.1080/17470210701438194 18938284

[14] Don HJ, Beesley T, Livesey EJ. Learned predictiveness models predict opposite attention biases in the inverse base-rate effect. J Exp Psychol Anim L 2019 45(2): 143–162. 10.1037/xan0000196 30869934

[15] Bourne LE Jr, Bunderson CV. Effects of delay of informative feedback and length of post-feedback interval on concept identification. J Exp Psychol 1963 65(1): 1–5. 10.1037/h0046994 14014497

[16] Bourne LE Jr, Guy DE, Dodd DH, Justeen DR. Concept identification: The effects of varying length and informational components of the intertrial interval. J Exp Psychol 1965 69(6): 624–629. 10.1037/h0022018 14304315

[17] Watson MR, Blair MR. (2008). Attentional allocation during feedback: Eyetracking adventures on the other side of the response. Cogsci 2008 30(30). https://escholarship.org/uc/item/66r3x539

[18] Worthy DA, Markman AB, Maddox WT. Feedback and stimulus-offset timing effects in perceptual category learning. Brain Cogn 2013 81(2): 283–293. 10.1016/j.bandc.2012.11.006 23313835 PMC3560315

[19] Maddox WT, Ashby FG, Bohil CJ. Delayed feedback effects on rule-based and information integration category learning. J Exp Psychol Learn Mem Cogn 2003 29(4): 650–662. 10.1037/0278-7393.29.4.650 12924865

[20] Smith JD, Boomer J, Zakrzewski AC, Roeder JL, Church BA, Ashby FG. Deferred feedback sharply dissociates implicit and explicit category learning. Psychol Sci 2014 25(2): 447–457. 10.1177/0956797613509112 24335605 PMC3946254

[21] Ashby FG, Queller S, Berretty PM. On the dominance of unidimensional rules in unsupervised categorization. Percept Psychophys 1999 61(6): 1178–1199. 10.3758/BF03207622 10497436

[22] Leong YC, Radulescu A, Daniel R, DeWoskin V, Niv Y. Dynamic interaction between reinforcement learning and attention in multidimensional environments. Neuron 2017 93(2): 451–463. 10.1016/j.neuron.2016.12.040 28103483 PMC5287409

[23] Gottlieb J. Understanding active sampling strategies: Empirical approaches and implications for attention and decision research. Cortex 2018 102: 150–160. 10.1016/j.cortex.2017.08.019 28919222 PMC5826782

[24] Barnes, JI, Blair MR, Tupper PF, Walshe RC. A dynamic neural field model of self- regulated eye movements during category learning. CogSci 2015 148–153.

[25] McColeman CM, Barnes JI, Chen L, Meier KM, Walshe RC, Blair MR. Learning-induced changes in attentional allocation during categorization: A sizable catalog of attention change as measured by eye movements. PLoS One 2014 9(1). 10.1371/journal.pone.0083302 24497915 PMC3908863

[26] Chen L, Meier KM, Blair MR, Watson MR, Wood MJ. Temporal characteristics of overt attentional behavior during category learning. Atten Percept Psychophys 2013 75(2): 244–256. 10.3758/s13414-012-0395-8 23151960

[27] Meier KM, Blair MR. Waiting and weighting: Information sampling is a balance between efficiency and error-reduction. Cognition 2013 126(2): 319–325. 10.1016/j.cognition.2012.09.014 23099124

[28] Maddox WT, Filoteo JV, Hejl KD, Ing AD. Category number impacts rule-based but not information-integration category learning: Further evidence for dissociable category-learning systems. J Exp Psychol Learn Mem Cogn 2004 30: 227–245. 10.1037/0278-7393.30.1.227 14736309

[29] Morel P. Gramm: Grammar of graphics plotting in Matlab. J Open Source Softw 2018 3(23): 568. 10.21105/joss.00568

[30] Stephens RG, Kalish ML. The effect of feedback delay on perceptual category learning and item memory: Further limits of multiple systems. J Exp Psychol Learn Mem Cogn 2018 44(9): 1397–1413. 10.1037/xlm0000528 29389182

[31] Ashby FG, Vucovich LE. The role of feedback contingency in perceptual category learning. J Exp Psychol Learn Mem Cogn 2016 42(11): 1731–1746. 10.1037/xlm0000277 27149393 PMC5097011

[32] Eskreis-Winkler L, Fishbach A. (2019). Not learning from failure—The greatest failure of all. Psychol Sci 2019 30(12), 1733–1744. 10.1177/0956797619881133 31702452

[33] Halff HM. Stimulus presentation after successes and errors in concept-identification. Am J Psychol 1975 88(3): 421–430. 10.2307/1421772

[34] Hebb DO. The organization of b; a neuropsychological theory. Wiley; 1949. isbn: B000XCLB6W.

[35] Le Pelley ME, Newell BR, Nosofsky RM. Deferred feedback does not dissociate implicit and explicit category-learning systems: Commentary on Smith et al. (2014). Psychol Sci 2019 30(9): 1403–1409. 10.1177/0956797619841264 31343955

