# Supplementary material for: The ubiquity of selective attention in the processing of feedback during category learning
Source: PLoS One. 2021 Dec 16;16(12):e0259517. doi: 10.1371/journal.pone.0259517 (PMC8675756; doi:10.1371/journal.pone.0259517)
Supplement: S1 Table — Includes results of best fitting LME models for all 6 research questions, including analysis 4.1 (additional under research question 4). (PDF) [file pone.0259517.s001.pdf]

analysis	exp	model	intercept	SE	effect 1	coefficient	SE	effect 2	coefficient	SE	effect 3	coefficient	SE
1	1	Duration ~ 1 + TrialBin + Phase + (1 + TrialBin | Subject)	4627.80	141.19	Phase	-919.19 (FB)	45.33	TrialBin	-366.57	20.30	N/A	N/A	N/A
1	2	Duration ~ 1 + Phase + Condition + TrialBin + (1 + TrialBin|Subject)	3453.10	297.42	Phase	285.13 (FB)	99.16	Condition	458.10 (1:1)	124.20	TrialBin	-162.81	19.55
1	3	Duration ~ 1 + Condition + TrialBin + Phase + (1 + TrialBin | Subject)	3125.30	108.03	Phase	-719.87 (FB)	57.47	Condition	257.75 (acc)	76.96	TrialBin	-195.88	9.94
1	4	Duration ~ 1 + TrialBin + Phase + (1 + TrialBin | Subject)	1808.60	130.77	Phase	-114.63 (FB)	47.32	TrialBin	-124.59	11.57	N/A	N/A	N/A
1	5	Duration ~ 1 + TrialBin + Phase + (1 + TrialBin | Subject)	3978.70	189.57	Phase	-1037.6 (FB)	53.43	TrialBin	-190.01	13.09	N/A	N/A	N/A
1	6	Duration ~ 1 + Condition + TrialBin + (1 + TrialBin | Subject)	2594.80	177.21	TrialBin	-220.77	20.97	Condition	-422.55 (RB)	91.11	N/A	N/A	N/A
2	1	Duration ~ 1 + AOI + (1 | Subject)	822.70	32.41	AOI	-357.8 (irrel); -379.3 (FB)	25.23	N/A	N/A	N/A	N/A	N/A	N/A
2	2	Duration ~ 1 + AOI + (1 | Subject)	1119.50	106.37	AOI	-298.90 (irrel); -27.83 (FB)	42.64	N/A	N/A	N/A	N/A	N/A	N/A
2	3	Duration ~ 1 + AOI + (1 | Subject)	803.360	78.31	AOI	-312.92(irrel); -374.57 (FB)	37.7	N/A	N/A	N/A	N/A	N/A	N/A
2	4	Duration ~ 1 + Condition + AOI + (1 | Subject)	1281.90	133.36	AOI	-377.29 (irrel); -619.24 (FB)	61.67	Condition	-480.72 (speed)	185.40	N/A	N/A	N/A
2	5	Duration ~ 1 + AOI + (1 | Subject)	546.38	46.93	AOI	-301.15(irrel);-139.29 (FB)	28.1	N/A	N/A	N/A	N/A	N/A	N/A
2	6	Duration ~ 1 + Condition + AOI + (1 | Subject)	446.04	49.32	AOI	-321.04 (irrel); -358.08 (FB)	27.42	Condition	431.36 (RB)	73.89	N/A	N/A	N/A
3	1	pFix ~ 1 + Rank + AOI + (1 | Subject)	0.3535	0.0104	AOI	'-0.08754 (irrel); 0.2754 (FB)	0.007355	Rank	-0.1063	0.006005	N/A	N/A	N/A
3	2	pFix ~ 1 + Rank + AOI + (1 | Subject)	0.5169	0.01226	AOI	-0.1572 (irrel); -0.1274 (FB)	0.008668	Rank	-0.1067	0.007077	N/A	N/A	N/A
3	3	pFix ~ 1 + Rank + AOI + (1 | Subject)	0.4052	0.01603	AOI	-0.1339 (irrel); -0.02468 (FB)	0.009262	Rank	-0.09525	0.007562	N/A	N/A	N/A
3	4	pFix ~ 1 + Condition + Rank + AOI + (1 | Subject)	0.5034	0.02739	AOI	-0.1569(irrel); -0.1790(FB)	0.01537	Rank	-0.09108	0.01255	Condition	-0.06280 (speed)	0.02475
3	5	pFix ~ 1 + Rank + AOI + (1 | Subject)	0.4401	0.01265	AOI	-0.1739 (irrel); 0.1249 (FB)	0.008946	Rank	-0.1268	0.007304	N/A	N/A	N/A
3	6	pFix ~ 1 + Condition + Rank + AOI + (1 | Subject)	0.5197	0.0166	AOI	-0.2548 (irrel); -0.09898 (FB)	0.01162	Rank	-0.1340	0.009490	Condition	0.04503 (RB)	0.01205
4	1	dist ~ 1 + TrialBin + (1 + TrialBin | Subject)	1617.10	59.00	TrialBin	-100.38	8.22	N/A	N/A	N/A	N/A	N/A	N/A
4	2	dist ~ 1 + Condition + TrialBin + (1 + TrialBin | Subject)	1112.40	76.81	Condition	246.28 (1:1)	59.38	TrialBin	-45.05	3.97	N/A	N/A	N/A
4	3	dist ~ 1 + Condition + TrialBin + (1 + TrialBin | Subject)	1377.70	72.39	Condition	211.10 (acc)	77.51	TrialBin	-61.64	5.74	N/A	N/A	N/A
4	4	dist ~ 1 + TrialBin + (1 + TrialBin | Subject)	1089.70	74.71	TrialBin	-40.51	6.56	N/A	N/A	N/A	N/A	N/A	N/A
4	5	dist ~ 1 + TrialBin + (1 + TrialBin | Subject)	1418.70	81.83	TrialBin	-67.30	5.42	N/A	N/A	N/A	N/A	N/A	N/A
4	6	dist ~ 1 + Condition + TrialBin + (1 + TrialBin | Subject)	1065.10	77.95	Condition	-125.49 (RB)	57.44	TrialBin	-86.02	8.19	N/A	N/A	N/A
5	1	fixDuration ~ 1 + TrialBin + Phase + AOI + (1 + TrialBin | Subject)	307.88	4.20	Phase	-40.80 (FB)	1.65	TrialBin	-6.30	0.4815	AOI	1.30 (irrel); -35.31 (FB)	1.80 (irrel); 2.10 (FB)
5	2	fixDuration ~ 1 + TrialBin + Phase + AOI + (1 + TrialBin | Subject)	339.25	9.86	Phase	43.14 (FB)	2.23	TrialBin	-3.82	0.4238	AOI	4.08 (irrel; not sig); -39.27 (FB)	2.74 (irrel); 2.72 (FB)
5	3	fixDuration ~ 1 + Phase + AOI + (1 | Subject)	394.15	19.83	Phase	-38.32 (FB)	12.62	AOI	-28.40 (irrel); -179.71 (FB)	13.71 (irrel); 17.41 (FB)	N/A	N/A	N/A
5	4	fixDuration ~ 1 + TrialBin + Phase + AOI + (1 + TrialBin | Subject)	452.14	29.94	Phase	-75.55 (FB)	18.87	TrialBin	11.60	3.59	AOI	-55.10 (irrel); -233.56 (FB)	22.46 (irrel); 30.80 (FB)
5	5	fixDuration ~ 1 + TrialBin + Phase + AOI + (1 + TrialBin | Subject)	291.90	7.89	Phase	-19.04 (FB)	3.1	TrialBin	-4.28	0.4745	AOI	-14.93 (irrel); 105.74 (FB)	3.24 (irrel); 3.92 (FB)
5	6	fixDuration ~ 1 + TrialBin + Phase + AOI + (1 + TrialBin | Subject)	319.68	7.52	Phase	-31.39 (FB)	3.97	TrialBin	-7.33	0.7725	AOI	-35.79 (irrel); -9.47 (FB)	4.30 (irrel); 4.86 (FB)
6	1	Duration ~ 1 + AOI + Accuracy + (1 | Subject)	1067.10	33.42	AOI	-384.18 (irrel); -421.56 (FB)	20.49	Accuracy	-343.16 (correct)	16.76	N/A	N/A	N/A
6	2	Duration ~ 1 + AOI + Accuracy + (1 | Subject)	1474.80	116.23	AOI	-332.81 (irrel); -16.84 (FB)	35.17	Accuracy	-368.84 (correct)	28.72	N/A	N/A	N/A
6	3	Duration ~ 1 + AOI + Accuracy + (1 | Subject)	919.28	78.96	AOI	-344.45 (irrel); -394.65 (FB)	29.65	Accuracy	-138.99 (correct)	24.21	N/A	N/A	N/A
6	4	Duration ~ 1 + Condition + AOI + Accuracy + (1 | Subject)	1507.30	139.70	AOI	-445.57 (irrel); -694.06 (FB)	54.73	Accuracy	-200.00 (correct)	44.85	Condition	-492.08 (speed)	194.43
6	5	Duration ~ 1 + AOI + Accuracy + (1 | Subject)	693.30	50.52	AOI	-350.10 (irrel); -197.14 (FB)	22.47	Accuracy	-143.37 (correct)	18.34	N/A	N/A	N/A
6	6	Duration ~ 1 + Condition + AOI + Accuracy + (1 | Subject)	534.23	54.31	AOI	-347.97 (irrel); -395.19 (FB)	23.36	Accuracy	-94.90 (correct)	19.08	Condition	461.75 (RB)	79.61
7	1	Clickthrough ~ 1 + TrialBin + (1 + TrialBin | Subject)	-0.009115 (not sig)	0.005320	TrialBin	0.03044	0.002136	N/A	N/A	N/A	N/A	N/A	N/A
7	2	Clickthrough ~ 1 + TrialBin + (1 + TrialBin | Subject)	0.01607	0.0053743	TrialBin	0.005571	0.0008943	N/A	N/A	N/A	N/A	N/A	N/A
7	3	Clickthrough ~ 1 + TrialBin + (1 + TrialBin | Subject)	0.1491	0.02480	TrialBin	0.04549	0.002643	N/A	N/A	N/A	N/A	N/A	N/A
7	4	Clickthrough ~ 1 + TrialBin + (1 + TrialBin | Subject)	0.4264	0.03724	TrialBin	0.03543	0.003228	N/A	N/A	N/A	N/A	N/A	N/A
7	5	Clickthrough ~ 1 + TrialBin + (1 + TrialBin | Subject)	0.03042	0.009840	TrialBin	0.01259	0.001846	N/A	N/A	N/A	N/A	N/A	N/A
7	6	Clickthrough ~ 1 + TrialBin + (1 + TrialBin | Subject)	0.1092	0.01882	TrialBin	0.02134	0.003110	N/A	N/A	N/A	N/A	N/A	N/A
4.1**	1	abs_diff ~ 1 +TrialBin + (1 + TrialBin | Subject)	0.1303	0.0038095	TrialBin	0.0037872	0.00069781	N/A	N/A	N/A	N/A	N/A	N/A
4.1**	2	abs_diff ~ 1 + TrialBin + (1 + TrialBin | Subject)	0.13166	0.0071425	TrialBin	0.0035255	0.0011832	N/A	N/A	N/A	N/A	N/A	N/A
4.1**	3	abs_diff ~ 1 + TrialBin + (1 + TrialBin | Subject)	0.18899	0.019493	TrialBin	-0.009232	0.0023313	N/A	N/A	N/A	N/A	N/A	N/A
4.1**	4	abs_diff ~ 1 + TrialBin + (1 + TrialBin | Subject)			TrialBin			N/A	N/A	N/A	N/A	N/A	N/A
4.1**	5	abs_diff ~ 1 + TrialBin + (1 + TrialBin | Subject)	0.13645	0.0066271	TrialBin	-0.0087554	0.0010351	N/A	N/A	N/A	N/A	N/A	N/A
4.1**	6	abs_diff ~ 1 + Condition + TrialBin + (1 + TrialBin | Subject)	0.26541	0.013735	TrialBin	-0.010248	0.0019538	Condition	-0.13052 (RB)	0.018671	N/A	N/A	N/A

**NOTE: analysis 4.1 is the additional analysis under research question 4, examining the proportion of time spent looking at irrelevant stimulus features
